# Effect of chronic treatment with Rosiglitazone on Leydig cell steroidogenesis in rats: *In vivo *and *ex vivo *studies

**DOI:** 10.1186/1477-7827-8-13

**Published:** 2010-02-09

**Authors:** Janaína A Couto, Karina LA Saraiva, Cleiton D Barros, Daniel P Udrisar, Christina A Peixoto, Juliany SB César Vieira, Maria C Lima, Suely L Galdino, Ivan R Pitta, Maria I Wanderley

**Affiliations:** 1 Department of Morphology and Physiology, Universidade Federal Rural de Pernambuco, Recife, 52.171-900, Brazil; 2 Ultrastructure Laboratory, Aggeu Magalhães Research Center (FIOCRUZ) and Center for Strategic Technologies of the Northeast (CETENE), Recife, 50.670-901, Brazil; 3 Department of Antibiotics, Universidade Federal de Pernambuco, Recife, 50.670-901, Brazil; 4 Department of Physiology and Pharmacology, Universidade Federal de Pernambuco, Recife, 50.670-901, Brazil

## Abstract

**Background:**

The present study was designed to examine the effect of chronic treatment with rosiglitazone - thiazolidinedione used in the treatment of type 2 diabetes mellitus for its insulin sensitizing effects - on the Leydig cell steroidogenic capacity and expression of the steroidogenic acute regulatory protein (StAR) and cholesterol side-chain cleavage enzyme (P450scc) in normal adult rats.

**Methods:**

Twelve adult male Wistar rats were treated with rosiglitazone (5 mg/kg) administered by gavage for 15 days. Twelve control animals were treated with the vehicle. The ability of rosiglitazone to directly affect the production of testosterone by Leydig cells *ex vivo *was evaluated using isolated Leydig cells from rosiglitazone-treated rats. Testosterone production was induced either by activators of the cAMP/PKA pathway (hCG and dbcAMP) or substrates of steroidogenesis [22(R)-hydroxy-cholesterol (22(R)-OH-C), which is a substrate for the P450scc enzyme, and pregnenolone, which is the product of the P450scc-catalyzed step]. Testosterone in plasma and in incubation medium was measured by radioimmunoassay. The StAR and P450scc expression was detected by immunocytochemistry.

**Results:**

The levels of total circulating testosterone were not altered by rosiglitazone treatment. A decrease in basal or induced testosterone production occurred in the Leydig cells of rosiglitazone-treated rats. The ultrastructural and immunocytochemical analysis of Leydig cells from rosiglitazone-treated rats revealed cells with characteristics of increased activity as well as increased StAR and P450scc expression, which are key proteins in androgen biosynthesis. However, a number of rosiglitazone-treated cells exhibited significant mitochondrial damage.

**Conclusion:**

The results revealed that the Leydig cells from rosiglitazone-treated rats showed significant reduction in testosterone production under basal, hCG/dbcAMP- or 22 (R)-OH-C/pregnenolone-induced conditions, although increased labeling of StAR and P450scc was detected in these cells by immunocytochemistry. The ultrastructural study suggested that the lower levels of testosterone produced by these cells could be due to mitochondrial damage induced by rosiglitazone.

## Background

Rosiglitazone is a PPARγ synthetic activator from the group of thiazolidinediones (TZDs) often used in the treatment of chronic diseases such as type 2 diabetes and other forms of insulin resistance, as seen in polycystic ovary syndrome (PCOS). Activation of PPARγ by TZDs improves insulin sensitivity and, consequently, bodily glycemia and lipid control and reduces the concentration of plasma androgen in patients with PCOS [1-5]. Besides their well-known effects on insulin sensitivity and energy metabolism, TZDs have also been reported to modulate steroid production in gonad tissues. For example, TZDs stimulate progesterone secretion in MA-10 Leydig tumor cells [6,7] and ovarian cells [8]. Opposing effects of TZDs on androgen levels and/or production in male humans [9-11] and animal models have been described [7,12-20]. An inhibitory effect of rosiglitazone on the production of testosterone has been demonstrated in healthy men [11], while in obese male Zucker rats [19] plasma testosterone was not affected by this TZD. TZDs have also been reported to directly inhibit steroidogenic enzymes and steroid secretion *in vitro*, as evidenced by a decrease in the activity of 17α-hydroxylase, 17,20-lyase, aromatase and 3β-hydroxysteroid dehydrogenase [7,12-18]. Although there is evidence of the modulation of testosterone action and/or production by TZDs, the effects of oral rosiglitazone treatment on circulating plasma testosterone and/or production have not been reported in experimental animal models.

The aim of the present study was to determine whether oral rosiglitazone treatment influences testicular production of testosterone using an *ex-vivo *model of Leydig cells isolated from rosiglitazone-treated adult male rats. Ultrastructural and immunocytochemical analysis of Leydig cell was performed to assess the cellular integrity and the expression of StAR and P450scc, which are key proteins in androgen biosynthesis.

## Methods

### Animals and experimental procedures

Twelve male Wistar-Albino rats (aged 8-9 weeks and weighing 200-250 g) were used and obtained from the Bioterium of the Department of Antibiotics, Universidade Federal de Pernambuco, Brazil. The rats were kept in a small colony in the animal house at a temperature of 22 ± 3°C, with a 12:12 hour light/dark cycle, receiving standard feed (Purina^®^) and water as required. The experimental procedure was approved by the local Ethics Committee for Animal Experimentation. The rats were randomly divided into two groups of six animals each. The test group received rosiglitazon (GlaxoSmithKline, Aranda de Duero, Spain) prepared using 1% (v/v) Tween-80 (Sigma Chemical Co., St. Louis, MO, USA) and administered daily via oral gavage at 5 mg/kg/d. The control group received 1% vehicle. Both groups were treated for 15 consecutive days. After treatment, the rats were sacrificed with carbon dioxide. Following decapitation, truncal blood was collected in heparinized tubes and kept on ice until the plasma was obtained by centrifugation (600 × g, 15 min. 6°C). The plasma was aliquoted and stored at -20°C until analysis for testosterone concentration. The testes and seminal vesicles were removed and weighed. The testes were used for *ex vivo *assays and morphological analysis. The dry weight of the seminal vesicles was determined by drying these tissues in an oven overnight at 110°C.

### *Ex vivo *study

For the *ex vivo *study, the testes from rosiglitazone-treated or control rats were decapsulated and the Leydig cells were isolated and purified as described in Wanderley and Negro-Vilar [21], with slight modifications. Briefly, the decapsulated testes were incubated in an enzyme solution of 0.5 mg/ml collagenase (Sigma), 0.2 mg/ml soybean trypsin inhibitor (Sigma) and 5 μg/ml leupeptin (Sigma) in PBS (136.9 mM NaCl, 2.68 mM KCl, 8.1 mM Na_2_HPO_4_.7H2O, 1.47 mM KH_2_PO_4_) containing 0.1% bovine serum albumin (PBS/BSA) (BSA, fraction V, ICN Biomedical, California, CA, USA), pH 7.4, in a shaking water bath (20 min, 90 Hz, 34°C). The dispersed testes were suspended in 50 ml (final volume) PBS/BSA and the dissociated tubules were allowed to settle (5 min). The supernatant was filtered and washed with 5 ml PBS/BSA. The filtered cell suspension was centrifuged (150 × g, 15 min, 20°C). The pellet was re-suspended in 5 ml PBS/BSA, loaded onto the top of a discontinuous Percoll (HE Healthcare Bio-Sciences AB, Uppsala, Sweden) density gradient (20%, 35%, 43%, 68% and 90%) and centrifuged at 800 × g for 30 min at 20°C. Cells in the 43-68% interface (specific gravity: 1.0640-1.0960 g/ml) were collected, washed twice with medium 199 (M199) (Gibco, Grand Island, NY, USA) containing 0.1% BSA, re-suspended in M199/0.1% BSA and used immediately for the experiments. Cells (0.3 × 10^6 ^cells/0.5 ml) were treated (incubated) for 2 h with M199 (basal testosterone), hCG (Sigma) (1 mIU/ml), dbcAMP (Sigma) (1 mM), 22-hydroxycholesterol (Sigma) (10 μM) or pregnenolone (Sigma) (1 μM) (stimulated/induced testosterone) in a shaking water bath (60 Hz, 34°C) in an atmosphere of 95% O_2 _and 5% CO_2_. At the end of incubation, the cells were centrifuged. The supernatant was collected and stored at -20°C until testosterone measurement by radioimmunoassay (RIA). To assess the effects of chronic treatment with rosiglitazone on cell viability the trypan blue exclusion was used. The trypan blue exclusion is widely used screening method to measure plasma membrane integrity. The trypan blue assay was performed after the period of 2 h incubation of cells. Cells were incubated with trypan blue (0,5%) for 20 min and the percentage of blue cells, indicating a capture of the colorant due to plasma membrane rupture, were counted. 90-95% of no colored cells was considered normal cell viability.

### Light microscopy

The testes were fixed in Bouin's solution for eight hours, then dehydrated in an alcohol series and embedded in paraffin wax. Serial sections of 4 μm were cut with a microtome (Leica RM 3125RT) and stained with hematoxylin-eosin for histological analysis [22].

### Electron transmission microscopy

For routines procedures, the fragments of testes were fixed overnight in a solution containing 2.5% glutaraldehyde (Sigma) and 4% paraformaldehyde (Sigma) in 0.1 M cacodylate (Sigma) buffer. After fixation, the samples were washed twice in the same buffer and post-fixed in a solution containing 1% osmium tetroxide (Sigma), 2 mM calcium chloride and 0.8% potassium ferricyanide in 0.1 cacodylate buffer, pH 7.2, dehydrated in acetone and embedded in SPIN-PON resin (Embed 812). Polymerization was performed at 60°C for three days [23]. Ultrathin sections were collected on 300-mesh nickel grids, counterstained with 5% uranyl acetate and lead citrate and examined with a FEI Morgani 268D transmission electron microscope. For the immunocytochemical study, the isolated and hCG-stimulated Leydig cells were fixed overnight in a solution containing 0.5% glutaraldehyde and 4% paraformaldehyde in 0.1 M phosphate buffer. After fixation, the samples were washed three times in the same buffer, incubated with 50 mM ammonium chloride for 40 min, dehydrated in alcohol and embedded in LR-White resin (Electron Microscopy Science, Washington, PA, USA). Polymerization was performed at 30°C for five days. This procedure was carried out as described by Peixoto et al. [24].

### Immunocytochemistry

Ultrathin sections of isolated and hCG-stimulated Leydig cells were cut with a diamond knife, collected on nickel grids and incubated for 30 min. at room temperature in 0.02 M PBS, pH 7.2, containing 1% BSA and 0.1% Tween 20 (PBS-BT). The sections were then incubated for one hour with primary antibodies against StAR and P450scc at dilutions of 1:25 and 1:200, respectively, in PBS-BT. The sections were then washed in PBS-BT and incubated with a secondary antibody, 10 nm colloidal gold-labeled goat anti-rabbit IgG. As an antibody control, sections were incubated only in the presence of the gold-labeled marker. Following the immunostaining procedures, the sections were counterstained with 5% uranyl acetate and lead citrate [24]. Quantitative analysis was performed on photomicrographs at a final magnification of 28'000× of 10 different Leydig cells, showing the entire profile and randomly chosen, in order to compare the number of gold-labeled particles in the control and rosiglitazone-treated cells using the Student's *t *test. Since the experimentally treated cells and control samples were processed in an identical method, no correction for tissue shrinkage was performed.

### Antibodies

The polyclonal antibodies StAR (sc-25806, Santa Cruz Biotechnology, INC., Santa Cruz, CA, USA) and cytochrome P450scc enzyme (AB1244, Chemicon International, Inc., Canada) were raised in rabbits against different peptides corresponding to amino acids 1-285 representing full-length StAR of human origin and amino acids 421-441 of the rat cytochrome P450scc enzyme, respectively. The 10-nm colloidal gold-labeled goat anti-rabbit IgG was purchased from Sigma Chemical Co. (St. Louis, MO, USA).

### Radioimmunoassay and statistical analysis

Testosterone was measured in plasma (with extraction) and directly (without extraction) in the incubation medium by a charcoal-dextran RIA [25] that employs [^3^H]-testosterone as a tracer and primary antiserum raised in rabbits in our laboratory against testosterone-3-(0-carboxymethyl)oxime:BSA. Intra-assay and inter-assay coefficients of variation were 8.1% and 15.1%, respectively. The testosterone antibody demonstrated < 0.1% cross-reactivity with androstenedione, dehydroepiandrosterone, androsterone, 17α-hydroxyprogesterone, β-estradiol and estrone. None of the substances tested interfered with the assays. The data from the different analyses are expressed as the mean ± SEM of six replicates determinations for plasma and triplicate determinations for incubation medium and were representative of results obtained in at least two similar experiments. The Student's *t *test, one-way analysis of variance (ANOVA) and Dunnett's test were used to determine differences between control and rosiglitazone-treated plasma and cells, respectively. P-values less than 0.05 were considered statistically significant.

## Results

### Body weight, testis weight, seminal vesicle weight and plasma testosterone

The treatment of rats with rosiglitazone did not induce changes in body weight or the relative weight of the testes or dried seminal vesicles (data not shown). Total plasma testosterone was not significantly modified by the treatment with rosiglitazone (control: 25.01 ± 3.5; rosiglitazone: 38.4 ± 7.0 ng/ml), which may explain the unaltered weight of the seminal vesicles, since it is known that testosterone supports the trophism of this organ.

### *Ex vivo *testosterone secretion

**Figure 1:**
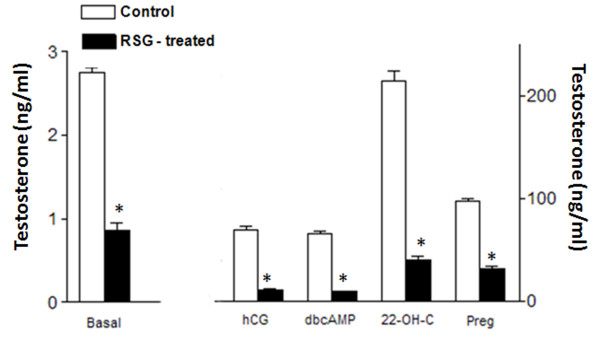
***Ex vivo *production of testosterone in Leydig cells isolated from control or rosiglitazone-treated (RSG-treated) rats; Leydig cells (0.3 × 10^6^/0.5 ml) were incubated for 2 h with M199, hCG (1 mIU/ml), dbcAMP (1 mM), 22-OH-C (10 μM) or pregnenolone (1 μM)**. Results are the mean ± SEM of three determinations; *p < 0.001 (ANOVA).

These experiments examined the impact of chronic treatment with rosiglitazone on the steroidogenic response of Leydig cells to direct induction by activators of the cAMP/PKA pathway (hCG and dbcAMP), which is the major signaling pathway regulating steroidogenesis [26], and by substrates of steroidogenesis (22-OH-C and pregnenolone). The objective of the use of these substrates was to determine whether the limiting-steps of steroidogenesis - the transportation of cholesterol from outer to inner mitochondrial membrane by the StAR protein and the cleavage of the cholesterol side chain by mitochondrial P450scc to yield pregnenolone - were affected by rosiglitazone treatment. Rosiglitazone treatment modified the steroidogenic response, resulting in a decrease in testosterone production under basal, hCG/dbcAMP- or 22 (R)-OH-C/pregnenolone-induced conditions (Figure 1). This reduced steroidogenic response to stimulators/inducers of testosterone production can be seen by the reduction in the magnitude of the stimulation/induction obtained in both the control and rosiglitazone-treated groups. The increase in testosterone production induced by hCG and dbcAMP in cells from control rats was 24.1-fold (± 1.2) and 22.9-fold (± 5.5), respectively, whereas the increase in production in cells from rosiglitazone-treated rats was 12.4-fold (± 0.4) and 10.5-fold (± 0.2), respectively. Similarly, the increase in testosterone production induced by 22(R)-OH-C and pregnenolone in cells from control rats was 77.0-fold (± 33.0) and 34.4-fold (± 0.9), respectively, whereas the increase in production in cells from rosiglitazone-treated rats was 46.0-fold (± 39.3) and 35.4-fold (± 20.2), respectively. These results indicate that the rosiglitazone treatment decreases the function of multiple steroidogenic enzymes, including P450scc. In order to evaluate whether the inhibitory effect of the chronic treatment with rosiglitazone on testosterone production was due to a decrease in cell viability, the trypan blue exclusion assay was used. This test demonstrated that inhibition was not due to the toxicity of rosiglitazone *per se*, as the percentages of integral cells obtained after 2 h of incubation were comparable between the cells from rosiglitazone-treated (93%) and from controls (95%) rats.

### Histological analysis

No morphological differences were found in Leydig cells between the control and experimental groups (data not shown).

### Electron microscopy analysis

**Figure 2:**
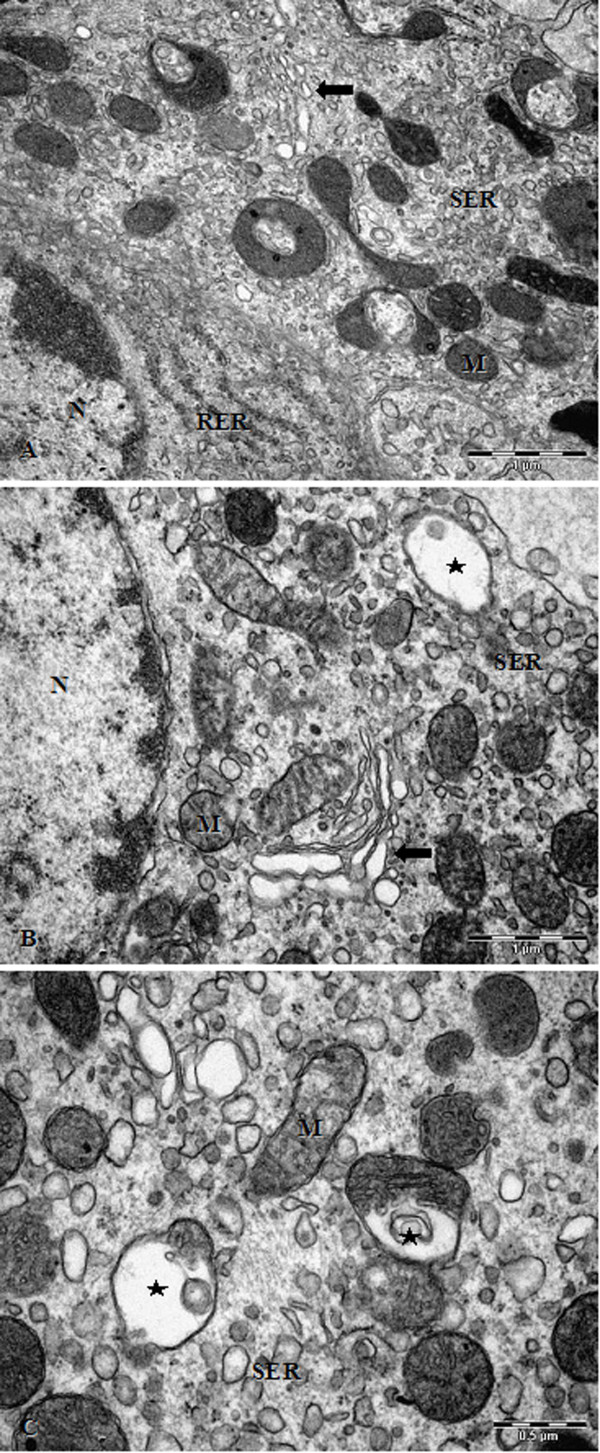
**Testis Leydig Cells; A - Untreated Leydig cells with nucleus, Golgi complex (arrows), SER, RER and mitochondria; Bar 1 μm; B and C - Leydig cells treated with rosiglitazone showing dilated and vacuolated mitochondria (star), vesicular smooth endoplasmic reticulum and dilated Golgi complex (arrows); Bars 1 μm and 0.5 μm, respectively; M, mitochondria; N, nucleus; RER, rough endoplasmic reticulum; SER, smooth endoplasmic reticulum**.

The morphological characteristics of control Leydig cells were an irregular polygonal shape with an asymmetrical nucleus located eccentrically in the cell body. The filopodia were protruded and interdigitated with the filopodia from opposite cells. The tubular smooth endoplasmic reticulum (SER) was abundant, consisting of interconnected, branched and anastomosing tubules throughout the cytoplasm. This tubular SER was occasionally surrounding mitochondria and lipid droplets. The rough endoplasmic reticulum (RER) was scarce, located mainly in perinuclear and peripheral regions. Mitochondria of variable size and with tubular and thick cristae were numerous and scattered throughout the cytoplasm. Several Golgi complexes were dispersed in the cell body, often found near a cell pole, since the nucleus is eccentrically located. Peroxisomes of irregular sizes were identified in portions of the cytoplasm (Figure 2:A). Rosiglitazone-treated Leydig cells presented all the organelles cited above. However, some differences were observed, such as a vesicular SER, consisting of separated vesicles of variable diameters and dilated Golgi complexes, which are typical alterations of an activated steroidogenic cell. However, several Leydig cells exhibited enlarged, vacuolated mitochondria with disarranged or discontinuous cristae (Figure 2:B and 2:C).

### StAR and P450scc in Leydig cells

**Figure 3:**
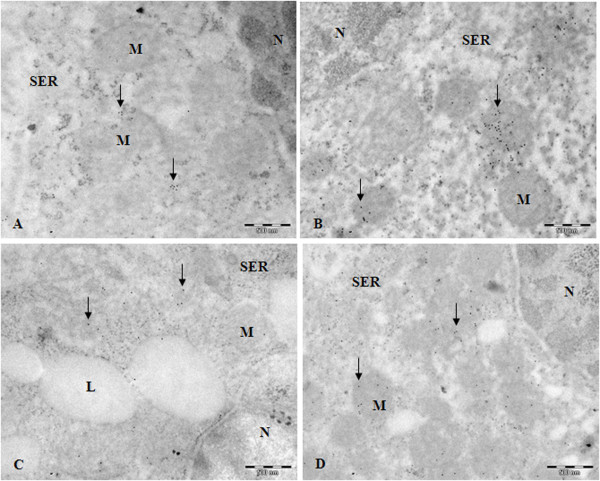
**StAR and P450scc immunocytochemistry; A and C - little labeling was observed in control samples for StAR (A) and P450scc (C) (arrows); B and D - increased labeling of StAR (B) and P450scc (D) was detected in rosiglitazone-treated Leydig cell (arrows); L, Lipid droplets; M, mitocondria; N, nucleus; SER, smooth endoplasmic reticulum; Bars 500 nm**.

**Figure 4:**
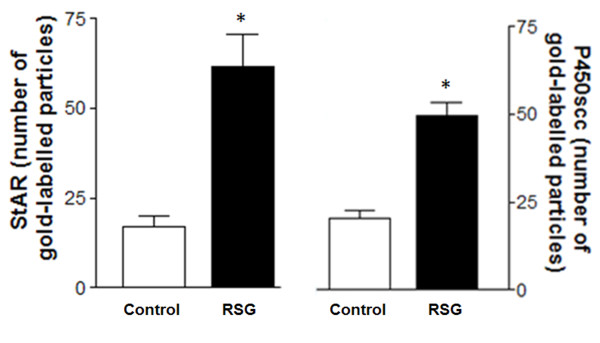
Quantitative analysis of gold-labeled particle distribution in control and rosiglitazone-treated (RSG) Leydig cells; Significant changes were found in treated cells (*Student's *t *test, p < 0.0002). Mean of gold-labeled particles (mean ± S.D); N = 10.

Immunocytochemistry revealed that the enzymatic steroidogenic pathway was significantly more active in the Leydig cells treated with rosiglitazone. More evident StAR and P450scc labeling was detected throughout the cytoplasm of treated cells in comparison to control samples (Figures 3 and 4).

## Discussion

Thiazolidinediones (TZDs) are frequently administered to patients with insulin resistance associated with type II diabetes [for a review, see [27-29]]. Moreover, there is growing evidence that the biological effects of TDZs go beyond insulin-sensitizing [30]. It has been demonstrated that TDZs have a considerable impact on the production and metabolism of gonad hormones [6,8,14,17-20]. Rosiglitazone and other thiazolidinediones are known to affect testosterone production in humans [10,11,31]. However, effects of rosiglitazone on testosterone level and synthesis have not been reported in experimental animal models. Therefore, the present study investigated the effect of chronic treatment with rosiglitazone on plasma testosterone levels, steroidogenic response and morphology of Leydig cells in normal rats.

Steroid hormones are synthesized from cholesterol in the gonads in response to pituitary hormones, such as LH/hCG via the classic cAMP/PKA pathway. The main rate-limiting step in the steroidogenenic pathway is the transportation of cholesterol from the outer to inner mitochondrial membrane by a transmembrane protein, StAR protein, the expression and activation of which is maintained by cAMP modulated PKA under maximal stimulation of LH [32].

In the present study, normal rats exposed to rosiglitazone underwent no changes in the level of total plasma testosterone when compared with the controls. A number of authors have reported contrasting results regarding the impact of TZDs on plasma testosterone levels in different clinical and experimental models. In male Zucker diabetic fatty (ZDP) rats, rosiglitazone treatment did not alter plasma testosterone levels [19,33]. In healthy men, rosiglitazone decreased testosterone and dihydrotestosterone (DHT) production rates [11]. In women with PCOS and hyperandrogenism, TZD treatment increases sex hormone binding globulin (SHBG) levels in plasma, leading to a decrease in free-circulating testosterone levels [34]. Circulating testosterone is known to be present in three major fractions: free, albumin-bound and sex hormone binding globulin (SHBG) [35]. In contrast to women, men with type 2 diabetes have low testosterone levels and treatment with rosiglitazone induces an increase in the three fractions of circulating testosterone and SHBG levels [36]. As the testosterone measured in the present study represents the total circulating fraction, the measurement of the other two fractions (free and albumin-bound) would be necessary for a better assessment of the effect of rosiglitazone treatment in the present study. However, unlike the human, adult rats do not express SHBG [37]. Additionally, the possibility of decreased clearance rate of testosterone can not be excluded.

In the *ex vivo *experiments, basal testosterone production was reduced by chronic treatment with rosiglitazone and Leydig cells from rosiglitazone-treated rats were less responsive to cAMP/PKA pathway activation of testosterone production than those from control rats. The *ex vivo *model is based on the ability of hCG/dbcAMP to stimulate testosterone production by Leydig cells *in vitro *through a mechanism involving PKA activation [26]. The results of the present study suggest a direct effect of rosiglitazone on basal and stimulated testosterone secretion on the PKA level and/or at a point downstream from PKA activation. To further study the earlier steps (before and after mitochondrial P450scc-catalyzed conversion of cholesterol into pregnenolone) of the steroidogenic pathway, 22 (R)-OH-C-induced and pregnenolone-induced testosterone production was also examined. 22 (R)-OH-C replaces cholesterol because it is membrane-permeable. The high concentrations used were to obviate the interference of any endogenous precursor. When Leydig cells from rosiglitazone-treated rats were challenged with 22 (R)-OH-C or pregnenolone, a reduction in testosterone production occurred, indicating that substrate (cholesterol) availability was not affected by the treatment with rosiglitazone, but rather multiple steroidogenic enzymes were limiting.

Morphological analysis of the testis was performed to determine whether the reduced testosterone production in the testes of rosiglitazone-treated rats was due to structural alterations in this tissue or a reduced amount of the proteins involved in the limiting steps of steroidogenesis - StAR and P450scc. Rosiglitazone-treated rats had Leydig cells with activated morphological characteristics, such as vesicular SER, dilated Golgi complexes and few or no lipid droplets. According to Ohata [38], after stimulation with gonadotropin, the inactive tubular SER is converted into vesicles of variable sizes and the author found either hypertrophy or dilatation of the Golgi complexes, which are both related to enhanced secretory cell function. Moreover, activated Leydig cells are characterized by few and small lipid droplets, since the cholesterol contained in the droplets is consumed for the synthesis of steroid. In the present study, rosiglitazone-treated Leydig cells exhibited some vacuolated, enlarged mitochondria distributed throughout cytoplasm. This altered mitochondrial morphology could lead to mitochondrial dysfunction. It has been demonstrated in rat and human hepatocytes, that thiazolidinediones cause mitochondrial dysfunction, followed by increased permeability, calcium influx, and nuclear condensation [39]. The order of toxicity by which thiazolidinediones cause mitochondrial dysfunction is troglitazone pioglitazone and rosiglitazone [40,41]. Although experimental studies on hepatocytes and isolated mitochondria have indicated that troglitazone but not rosiglitazone or pioglitazone induces mitochondrial dysfunction [39,40], it is possible that the chronic exposure to rosiglitazone, as used in the present study, could lead to mitochondrial damage. It has been proposed by Feinstein et al. (2005) that this effect of thiazolidinediones are PPARγ-independent and results from a direct interaction of the drugs with mitochondria, altering bioenergetics and potentially increasing reactive oxygen species production and causing ATP depletion. Recently, the importance of intact mitochondria with active respiration for LH-mediated Leydig cell steroidogenesis has been showed [41,42]. These authors showed that maintenance of mitochondrial membrane potencial, mitochondrial ATP synthesis, and mitochondrial pH are all required for acute steroid biosynthesis. The observed alteration in the state of mitochondria induced by rosiglitazone in our experiments could explain the lower levels of testosterone produced by these cells.

Protein expression levels were evaluated through immunocytochemistry. Leydig cells from rosiglitazone-treated animals exhibited more expressive immunolabelling for StAR and P450scc when compared to control samples. These results are apparently contradictory to the reduced testosterone production by Leydig cells found in the *ex vivo *experiments. However, studying human ovarian cells in cultures, Seto-Young et al. [43] found that rosiglitazone and pioglitazone stimulated the expression of PPARγ, insulin receptor, IRS-1 and StAR, in contrast to results obtained by the authors in a previous report [44], in which thiazolidinediones stimulated progesterone production, but inhibited estradiol and testosterone production in ovarian cells. Recently, other authors found that rosiglitazone stimulated StAR expression but did not affect estradiol and progesterone production by human ovarian cells [45]. The morphological analysis of rosiglitazone-treated testes in the present study revealed important structural damage in the mitochondria of Leydig cells. This alteration could affect (reduce) the function of the StAR and the steroidogenic enzyme P450scc located in the mitochondria, even at high levels of expression of both proteins. It is also possible that other enzymes of the steroidogenic process are affected by rosiglitazone. Previous *in vitro *studies have demonstrated that thiazolidinediones inhibit the activity of 17α-hydroxylase/17,20-lyase [7,12,14,15], which are key enzymes in human androgen synthesis, and aromatase [13].

Rosiglitazone is a ligand for PPARγ. Recently, the expression of PPARγ has been demonstrated in Leydig cells and an increase in PPARγ mRNA and protein levels has been observed in these cells after chronic treatment with glitazones [33,46]. Therefore, it seems reasonable that the expression of StAR and P450scc observed in the present study may be mediated by the nuclear receptor PPARγ. However, the mitochondrial damage could indicate that the influence of rosiglitazone on testosterone production could also be PPARγ-independent.

## Conclusion

In summary, the present study was designed to examine the effect of chronic treatment with rosiglitazone on the steroidogenic capacity of Leydig cells in normal adult rats. The results revealed that the Leydig cells from rosiglitazone-treated rats showed significant reduction in testosterone production under basal, hCG/dbcAMP- or 22 (R)-OH-C/pregnenolone-induced conditions, although increased labeling of StAR and P450scc was detected in these cells by immunocytochemistry. The ultrastructural study suggested that the lower levels of testosterone produced by these cells could be due to mitochondrial damage induced by rosiglitazone. Further studies are necessary to evaluate the impact of chronic treatment with rosiglitazone on the activity of the StAR and steroidogenic enzymes involved in testosterone production. Although the present study does not permit distinguishing a pituitary gland from the direct effect of rosiglitazone, the observation that this TDZ reduces testosterone production in an *ex vivo *model indicates that rosiglitazone has direct effects on Leydig cells that are independent of the effects of the drug on the secretion of gonadotropin. Finally, rosiglitazone action in testicular steroidogenesis is potentially of physiological and pathophysiological significance.

## Competing interests

The authors declare that they have no competing interests.

## Authors' contributions

JAC, CDB, DPU, JSBCV, MIW carried out the treatment of animals, Leydig cells isolation, radioimmunoassay and performed the statistical analysis. KLAS, CAP carried out the ultrastructural analysis and immunocytochemistry. MIW and CAP conceived of the study, and participated in its design and coordination. MIW, DPU, CAP, KLAS, JAC, CDB, JSBCV, MCL, SLG and IRP participated in the interpretation and analysis of the data and helped to draft the manuscript. All authors read and approved the final manuscript.
